# Exploring the Life Cycle of *Bactrocera latifrons*: A Detailed Age-Stage, Two-Sex Life Table

**DOI:** 10.3390/insects16020132

**Published:** 2025-01-29

**Authors:** Yutong Zhai, Xianru Zeng, Dewei Wei, Xiaodong Jiang, Xiuzhen Long, Zhan He, Yonghao Yu, Xuyuan Gao

**Affiliations:** 1 Key Laboratory of Green Prevention and Control on Fruits and Vegetables in South China Ministry of Agriculture and Rural Affairs, Nanning 530007, China; zaak654@163.com (Y.Z.); zxr@gxaas.net (X.Z.); wdw@gxaas.net (D.W.); nkyjxd@163.com (X.J.); longxiuzhen2006@163.com (X.L.); happy-hezhan@163.com (Z.H.); 2 Guangxi Key Laboratory of Biology for Crop Diseases and Insect Pests, Nanning 530007, China; 3 Plant Protection Research Institute, Guangxi Academy of Agricultural Sciences, Nanning 530007, China

**Keywords:** pepper fruit fly, developmental duration, male and female lifespan table, eggplant, group rearing

## Abstract

*Bactrocera latifrons* (Hendel) is a serious pest requiring quarantine in many countries worldwide. It has caused huge economic losses to the cultivation of solanaceous plants, such as peppers and eggplants. In this study, we constructed an age-stage, two-sex life table using eggplant as the host to clarify the occurrence and damage patterns of *B. latifrons* on this crop for better field prevention and control in the laboratory. The results showed that the egg, larval, and pupal stages lasted 4.3, 11.3, and 9.3 days, respectively. The average lifespan of adult females and males was 101 and 102 days, respectively. The egg hatching rate, larval survival rate, and pupal emergence rate were 96%, 88%, and 84%, respectively. The average generation time was 43.96 days, with an intrinsic rate of increase of 0.097 d^−1^ and a net reproductive rate of 73.4. We established the life table based on group rearing. The research findings provide essential data for rearing *B. latifrons* in the laboratory, offer a reference for the dynamic prediction of field population dynamics, and offer a theoretical foundation for developing pest control strategies for *B. latifrons*.

## Simple Summary

*Bactrocera latifrons* has caused great losses to peppers, eggplants, and tomatoes, which have always been favored by people as fruits and vegetables. In recent years, this pest has been intercepted in fruits and vegetables imported from other countries at many ports in China, indicating that the risk of *B. latifrons* spreading along with its hosts is very high, and thus should attract wide attention. To conduct a risk assessment of *B. latifrons*, it was group-reared with eggplant as the host, and an age-stage two-sex life table was adopted to explore the growth and development indexes and population parameters of *B. latifrons*. The results showed that the egg, larval, and pupal stages lasted 4.3, 11.3, and 9.3 days, respectively. The average lifespan of adult females and males was 101 and 102 days, respectively. The egg hatching rate, larval survival rate, and pupal emergence rate were 96%, 88%, and 84%, respectively. The average generation time was 43.96 days, with an intrinsic rate of increase of 0.097 d^−1^ and a net reproductive rate of 73.4. This study provides a basis for the monitoring of *B. latifrons* and the formulation of effective control strategies.

## 1. Introduction

The pepper fruit fly, *Bactrocera latifrons* (Hendel), is originally native to the Asia–Pacific region, but has recently spread to other tropical areas worldwide through global trade [1,2,3]. *B. latifrons* infests a wide variety of host plants, including 59 species across 14 plant families, primarily within the Solanaceae and Cucurbitaceae families. The infestation rate is higher in Solanaceae hosts than in Cucurbitaceae hosts. The key crops affected include peppers (*Capsicum annuum* L.), eggplants (*Solanum melongena* L.), and tomatoes (*Solanum lycopersicum* L.) [4]. Adult female *B. latifrons* lay their eggs in the fruit of host plants. Upon hatching, the larvae feed on the fruit tissues, causing rotting and fruit drop, which negatively impact fruit yield and quality, leading to serious economic losses [5]. Understanding the ecological characteristics of *B. latifrons* is crucial for effective pest management.

Understanding population ecology requires extensive quantitative data, including life-table statistics for the target species [6]. A life table provides a comprehensive description of a population’s development, survival, fertility, and life expectancy, and is commonly used to predict population growth [7]. However, the life-table data collected under natural conditions can be influenced by various environmental factors. As such, life-table data must be collected under controlled laboratory conditions to enable accurate assessments of the biological potential of pest populations while minimizing the effects of these factors [8].

Traditional age-specific life tables typically focus on female populations only [9,10,11,12]. However, the males and females of many insect species exhibit different development rates. Ignoring these sex differences can lead to inaccuracies in key population parameters, such as the intrinsic rate of increase, net reproductive rate, and mean generation time [13,14]. The age-stage, two-sex life table is an improvement over traditional methods, incorporating both sex and stage differentiation and providing a more comprehensive and reliable description of a population’s development, survival, fertility, and life expectancy [15,16].

Most studies of fruit fly life tables have used individually reared specimens [17,18,19]. However, fruit flies typically occur in clusters in natural settings. The life histories of insects reared individually and in groups substantially differ. For instance, group-reared *Pararge aegeria* (Lepidoptera: Nymphalidae) are smaller and females lay more eggs than individually reared ones [20,21]. Similarly, *Bupalus piniarius* (Lepidoptera: Geometridae) larvae grow faster in groups than individually [22], and *Propylea dissecta* (Coleoptera: Coccinellidae) differs in growth rate, preoviposition period, and adult body size with rearing method [23].

We conducted a series of preliminary experiments with different potential hosts in the early stage of our research. These potential hosts included tomatoes and peppers, in addition to eggplant. The results of the preliminary experiments showed that *B. latifrons* exhibited a high preference and adaptability to eggplant. After comprehensive consideration and evaluation, we selected eggplant as the host. We used eggplant as the host and adopted the group-rearing method to construct an age-stage, two-sex life table to gain a deeper understanding of the growth, development, and reproduction of *B. latifrons*. The aim of this approach was to elucidate the reproductive potential and population dynamics of *B. latifrons*. The findings are required for developing effective pest control strategies and offer scientific insights for agricultural production. Additionally, the group-rearing method presents a practical and efficient method of constructing life tables, saving time, space, and labor while approximating natural conditions. This method is a considerable advancement in insect population ecology and provides valuable predictions for future insect populations [24].

## 2. Materials and Methods

### 2.1. Insect Colony

Damaged peppers were collected from a pepper garden in Wuming District, Nanning City, Guangxi (23.159219′ N, 108.274831′ E), and subsequently housed in the insect-rearing facility of the Plant Protection Institute, Guangxi Academy of Agricultural Sciences. After the larvae in the damaged fruits developed into adults, they were identified as *B. latifrons* based on their morphological characteristics. The identification process was carried out following standard taxonomic keys and reference materials [25]. The identified adults were transferred to insect-rearing cages with a length, width, and height of 35 cm each. Ripe eggplants (*Solanum melongena* L.) were placed into the cages for oviposition once the adult insects reached sexual maturity. The eggplants were removed after 24 h, and the larvae were separately reared. Additional eggplants were provided as needed based on the number of larvae. Mature (third-instar) larvae were collected and placed in glass bottles filled with sand for pupation. The adults that emerged from these pupae formed the first generation in the laboratory. Using this method, multiple generations were continuously reared to establish a stable *B. latifrons* colony with eggplant as the host. The diet consisted of an equal mass ratio of yeast powder and sucrose. To approximate the natural environmental conditions for *B. latifrons*, the rearing conditions were set as follows: a temperature of 28 ± 2 °C, a relative humidity of 65 ± 5%, and a photoperiod of 14L:10D.

### 2.2. Observation of Developmental Duration and Survival Rate of B. latifrons Eggs, Larvae, and Pupae

Fifty eggs laid on the same day were randomly collected and placed on eggplant slices in Petri dishes with filter paper at the bottom. This procedure was repeated three times. The hatching of the eggs was observed and recorded daily at 20:00. Newly hatched first-instar larvae were transferred to Petri dishes containing fresh eggplant slices for rearing. Fresh eggplant slices were added daily to ensure an adequate food supply for the larvae. The developmental progress and survival of the larvae were also monitored. Before pupation, mature larvae were transferred to 250 mL jars containing a 4 cm layer of fine sandy soil (sand/soil ratio = 1:2, with 8–10% moisture content) at the bottom for pupation. The development, survival, and number of emerging adults were observed and recorded until no more adults emerged.

### 2.3. Observation of B. latifrons Adult Longevity and Fecundity

Emerging *B. latifrons* adults were paired for rearing, with 5 pairs per group, and placed in insect-rearing cages. A total of 8 replicates were used. Fresh eggplant slices were placed in Petri dishes at the bottom of the cages to allow the adults to lay eggs. Their diet consisted of yeast powder and sucrose in a 1:1 mass ratio. Every day at 20:00, the eggplant slices in the Petri dishes were replaced. During this period, the survival rate of the adults and their daily fecundity were monitored and recorded until all adults died.

### 2.4. Establishment and Analysis of the Life Table of the Experimental Population

The survival rate, female fecundity, and the longevity of both female and male adults of *B. latifrons* were determined using the parameters obtained from the developmental duration of each stage (as described in Section 2.2 and Section 2.3); TWOSEX-MSChart Ver. 10/4/2024. [26] was used to construct the two-sex life table for *B. latifrons*. This allowed for a systematic analysis of population dynamics and the calculation of key life-table parameters, including age-stage-specific survival rate (*S_xj_*), population age-specific survival rate (*l_x_*), age-stage-specific fecundity of female adults (*f_xj_*), population age-specific fecundity (*m_x_*), population age-specific net fecundity (*lxmx*), age-stage-specific life expectancy (*exj*), age-stage-specific reproductive value (*υ_xj_*), net reproductive rate (*R*_0_), intrinsic rate of increase (*r*), finite rate of increase (*λ*), and mean generation time (*T*).

### 2.5. Data Analysis

Data were statistically analyzed using Excel 2021 to calculate the mean values and standard errors. One-way ANOVA, conducted with IBM SPSS 24, was used to analyze the variance in developmental duration, fecundity, and other parameters of *B. latifrons*. Mean values were compared using Duncan’s new multiple range test. The data were analyzed according to the principles of the age-stage, two-sex population life table, and each life-table parameter was statistically examined using TWOSEX-MSChart Ver. 10/4/2024. Charts were generated using OriginPro 2021b SR1 v9.8.5.204 x64.

## 3. Results

### 3.1. Developmental Duration of Each B. latifrons Life Stage

The egg, larval, and pupal stages of *B. latifrons* lasted 4.3, 11.3, and 9.3 days, respectively. The sex ratio of the population was nearly 1:1 according to the number of males and females observed. The adult preoviposition period (APOP), defined as the time from adult emergence to the first oviposition, was 9 days. The total preoviposition period (TPOP), which refers to the time from egg hatching to the first oviposition, was 34 days (Table 1).

### 3.2. Age-Stage-Specific Survival Rate of B. latifrons

**Figure 1:**
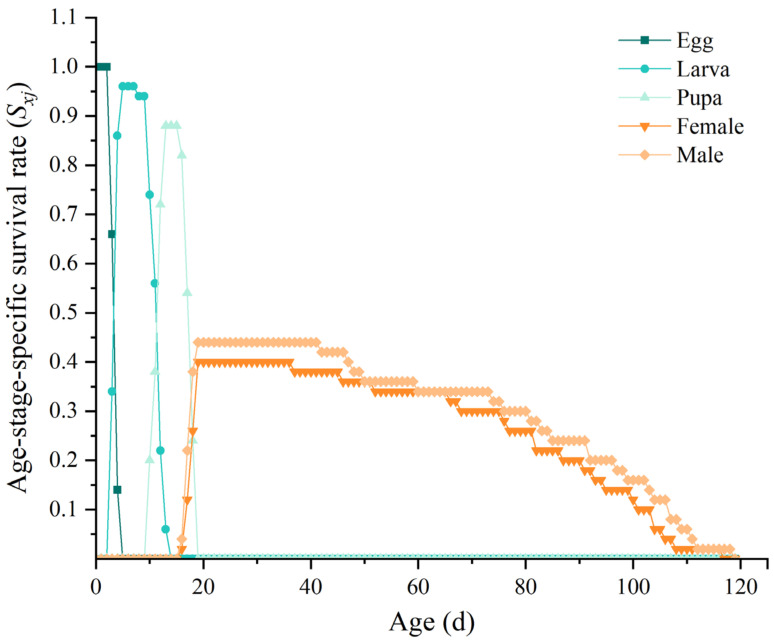
Age-stage-specific survival rate (*S_xj_*) of *Bactrocera latifrons*.

The age-stage-specific survival rate (*S_xj_*) represents the probability that an individual survives to age *x* and develops to stage *j* (Figure 1). Stage survival curves may overlap due to differences in individual development [27]. All *B. latifrons* eggs hatched by the fifth day of rearing, and the larval survival rate was 96%. The survival rate during the pupal stage was 88%. All pupae had emerged as adults by the 19th day of rearing, with an emergence rate of 84%. Among the emerged adults, 40% were female and 44% were male.

### 3.3. Age-Specific Survival Rate and Fecundity of B. latifrons

**Figure 2:**
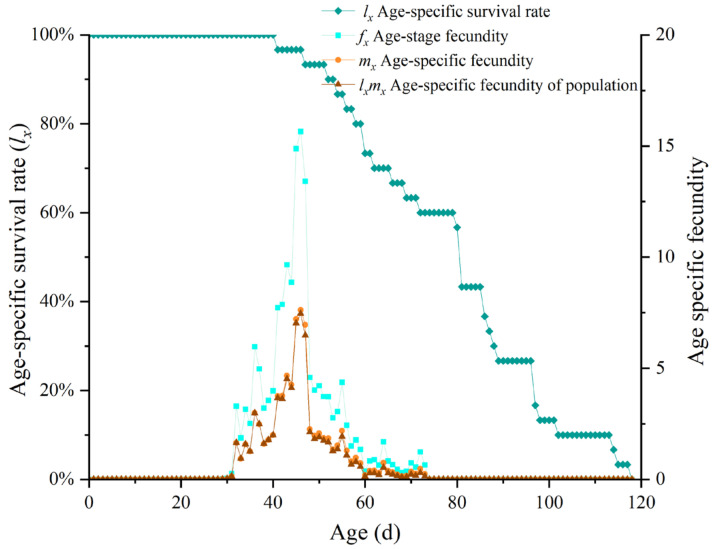
Age-specific survival rate and fecundity of *Bactrocera latifrons*.

The population age-specific survival rate (*l_x_*) represents the proportion of individuals surviving to age *x*. The analysis (Figure 2) showed that the *l_x_* curve steadily declined during the middle stage and rapidly dropped in the later stages, indicating a sharp decrease in the survival rate of adults in the later stages of life. The age-stage fecundity of female insects (*f_x_*) represents the fecundity of individual female *B. latifrons* at age *x*. The *f_x_* data indicated that female *B. latifrons* began oviposition on the 29th day and ceased laying eggs on the 75th day. The oviposition period lasted 45 days, with peak egg-laying occurring on the 46th day. The population age-specific fecundity (*m_x_*) represents the average fecundity of the entire population at age *x*, and the peak fecundity was 7.62 eggs.

### 3.4. Age-Stage-Specific Life Expectancy and Age-Stage-Specific Reproductive Value of B. latifrons

**Figure 3:**
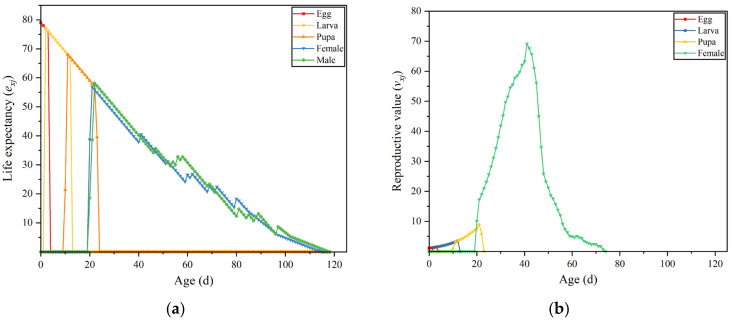
*Bactrocera latifrons* (**a**) age-stage-specific life expectancies (*e_xj_*) and (**b**) age-stage-specific reproductive value (*υ_xj_*).

The age-stage-specific life expectancy (*e_xj_*) represents the number of days an individual at age *x* and stage *j* is expected to survive. The analysis (Figure 3a) showed that life expectancy decreases with age, with the highest life expectancy observed in the egg stage, at 79.07 days. The age-stage-specific reproductive value (*υ_xj_*) indicates the contribution of an individual at age *x* and stage *j* to the future population. The analysis (Figure 3b) revealed that the reproductive value of *B. latifrons* increased as the insects progressed through the developmental stages, peaking at 68.19 on the 41st day. This suggests that the adult stage contributes the most to the future population.

### 3.5. Population Parameters of B. latifrons

The fecundity (*F*) of *B. latifrons* was 146.89, the net reproductive rate (*R*_0_) was 73.4, the intrinsic rate of increase (*r*) was 0.097 d^−1^, the mean generation time (*T*) was 43.96 days, and the finite rate of increase (*λ*) was 1.102 d^−1^ (Table 2).

## 4. Discussion

The pepper fruit fly *B. latifrons* is a pest that makes it necessary for plants imported to China and many other countries worldwide to be quarantined. This species is included in the EPPO A1 quarantine pest list, and its distribution is expanding [1,28]. *B. latifrons* has a relatively narrow host range compared with other fruit fly species, primarily affecting Solanaceae plants. This insect has resulted in serious economic losses in the Solanaceae crop industry [4,29]. This study is the first to present a detailed population life table for *B. latifrons* to provide a reference for integrated pest management.

The life table is a crucial tool in population ecology, pest management, and the study of species interactions, revealing insights into species adaptability [30]. In this study, 50 eggs were selected to explore the developmental duration and survival rates of *B. latifrons* eggs, larvae, pupae, and adults. The life-table analysis included both individual and group rearing methods. Previous studies have highlighted the differences between these rearing methods. The results showed that group-reared populations provided more reliable data for simulating pest population growth and management. Group rearing data are more reflective of natural population dynamics than individual rearing data, where gregarious species grow more slowly and exhibit higher mortality rates [31,32,33,34]. The life-table data from individually reared insects do not accurately reflect the characteristics of field populations because *B. latifrons* naturally occur in clusters. Group rearing, which more closely mimics natural conditions, is therefore a more appropriate method. However, counting subsequent egg laying was challenging due to the large number of adults emerging from the 50 eggs. Consequently, five pairs of adults were selected to study adult lifespan and fecundity, completing a full age-stage, two-sex life table for *B. latifrons*. Chi et al. demonstrated that the net reproductive rate (*R*_0_) is related to the average female fecundity (*F*) ads *R*_0_ = *F* × (*Nf*/*N*), where *N* is the total number of eggs used and *Nf* is the number of emerged female adults [30]. In this study, the group-rearing data satisfied this formula.

Larval stage parameters are critical indicators of the direct damage caused by pests during the insect life cycle [35]. *B. latifrons* larvae heavily feed on their host, causing substantial economic damage to Solanaceae crops. The larval stage lasts 11.3 ± 0.3 days at 28 °C. We compared the larval duration of several fruit fly species reared on various hosts at temperatures between 25 and 28 °C. For *Bactrocera dorsalis* (Diptera: Tephritidae), the larval stage lasts 7.84 ± 0.15 days on oranges, 8.13 ± 0.08 days on peaches, and 8.12 ± 0.11 days on apples. For *Bactrocera cucurbitae* (Diptera: Tephritidae), the larval stage lasts 7.4 ± 0.2 days on cucumbers, 7.4 ± 0.1 days on loofahs, and 8.5 ± 0.2 days on carrots. For *Bactrocera carambolae* (Diptera: Tephritidae), the larval stage lasts 10.6 ± 0.29 days on grapes and 9.2 ± 0.17 days on acerolas. In contrast, the larval stage of *B. latifrons* is substantially longer, damaging its host for a longer duration, leading to larger economic losses. Thus, timely pest management measures are essential. We acknowledge that there is a limitation in this study, namely the specific characteristics and classification criteria for each larval instar were not clearly defined, which is indeed a shortcoming of this study. However, the focus of this study was on constructing the age-stage, two-sex life table of *B. latifrons*, with the main concern being its overall population dynamics. This includes key parameters such as survival, development time, and reproduction at each stage from egg to adult. The classification of larval instars was not a core element for achieving this primary goal. Subsequent research can build on this study and conduct an in-depth exploration of the definition of larval instars to further improve our understanding of the biological characteristics of *B. latifrons*.

The adult stage is the longest in the complete life cycle of *B. latifrons*. The adult lifespan of females and males was 101.0 ± 0.6 days and 102.0 ± 1.5 days, respectively, with a 45-day oviposition period. Previous studies summarized the development times of adult females and males of *B. dorsalis*, *B. cucurbitae*, and *B. carambolae* within the 25–28 °C range. Female lifespans range from 55.4 ± 3.4 days to 88.1 ± 5.81 days, and male lifespans range from 50.1 ± 5.6 days to 84.0 ± 10.0 days [8,35,36]. These results indicate that within this temperature range, *B. latifrons* has the longest survival time. Additionally, the oviposition period of *B. dorsalis* ranges from 52.5 ± 3.0 to 66.0 ± 3.43 days; for *B. carambolae*, it ranges from 46.8 ± 14.2 to 60.3 ± 4.87 days [35,36]. In comparison, the oviposition period of *B. latifrons* is relatively shorter—less than half of its adult lifespan. Thus, although *B. latifrons* has a long adult stage, the adults are less harmful during this period than *B. dorsalis* and *B. carambolae*. Understanding the lifespan and reproduction cycle of adult insects can help determine the interval between multiple pesticide applications. For example, to prevent adults from continuously laying eggs and hatching new larvae that damage crops, based on the lifespan and oviposition period of adults, the re-application of pesticides can be carried out within a certain period after the peak of adult emergence, thus disrupting the pest’s reproduction chain.

Understanding the population dynamics of invasive pests and their potential growth is crucial for developing effective pest control strategies [37]. The population parameters obtained in this study provide valuable data for predicting field population dynamics. The population parameters calculated through the life table can predict the population growth trends of *B. latifrons* in the field. This is conducive to formulating prevention and control plans in advance, rationally arranging human, material, and pesticide resources, and avoiding economic losses caused by large-scale outbreaks of pests. After implementing pest control measures, by using the life-table data to compare the survival, development, and reproduction of pests before and after the control, the effectiveness of the control measures can be accurately evaluated. If it is found that after the implementation of a certain control measure, the mortality rate of pests does not meet expectations or the reproduction rate remains high, the reasons can be analyzed and the control strategy can be adjusted based on the information provided by the age-stage, two-sex life table. However, this study was conducted using eggplant as the host under specific conditions (temperature: 28 ± 2 °C; relative humidity: 65 ± 5%; photoperiod: 14L:10D), which may limit the generalizability of the results. Further investigation is needed to determine how the age-stage, two-sex life table of *B. latifrons* varies with different hosts, temperature–humidity conditions, and photoperiods.

## 5. Conclusions

We constructed the age-stage, two-sex life table of *B. latifrons* via group rearing for the first time and determined the growth and development indicators and population parameters of *B. latifrons*. The egg, larval, and pupal stages and the longevity of adult female and male *B. latifrons* were 4.3, 11.3, 9.3, 101, and 102 days, respectively. The egg hatching, larval survival, and pupal emergence rates were 96%, 88%, and 84%, respectively. The mean generation time of *B. latifrons* on eggplant was 43.96 days, the intrinsic rate of increase was 0.097 d^−1^, and the net reproductive rate was 73.4. These data are theoretically and practically valuable for formulating effective prevention and control strategies. Using group rearing to study the life tables of insect populations is a further innovation based on the research of age-stage, two-sex life tables, and is vital for predicting future insect populations.

## Figures and Tables

**Table 1:** The developmental durations of *Bactrocera latifrons*.

Stage (Days)	*n*	$\bar{x}$ ± SE
Egg	50	4.3 ± 0.3
Larva	47	11.3 ± 0.3
Pupa	44	9.3 ± 0.3
Adult (Female)	20	101.0 ± 0.6
Adult (Male)	22	102.0 ± 1.5
Adult Preoviposition Period	22	9.0 ± 0.6
Total Preoviposition Period	22	34.0 ± 1.0

**Table 2:** Population parameters (mean ± SE) of *Bactrocera latifrons*.

Population Parameter	Mean ± SE
Fecundity (*F*)	146.89 ± 19.41
Net reproductive rate (*R*_0_)	73.40 ± 9.71
Intrinsic rate of increase (*r*) (d^−1^)	0.097 ± 0.005
Finite rate of increase (*λ*) (d^−1^)	1.102 ± 0.005
Mean generation time (*T*) (d)	43.96 ± 0.77

## Data Availability

The original data are included in this article; further inquiries can be directed to the corresponding author.
